# CUI-Net: a correcting uneven illumination net for low-light image enhancement

**DOI:** 10.1038/s41598-023-39524-5

**Published:** 2023-08-09

**Authors:** Ke Chao, Wei Song, Sen Shao, Dan Liu, Xiangchun Liu, XiaoBing Zhao

**Affiliations:** 1 School of Information Engineering, Minzu University of China, Beijing, 100081 China; 2 Key Laboratory of Marine Environmental Survey Technology and Application, Ministry of Natural Resource, Guangzhou, 510300 China; 3 Language Information Security Research Center, Institute of National Security MUC, Minzu University of China, Beijing, 100081 China; 4 National Language Resource Monitoring and Research Center of Minority Languages, Minzu University of China, Beijing, 100081 China; 5 Key Laboratory of Ethnic Language Intelligent Analysis and Security Governance of MOE, Minzu University of China, Beijing, China

**Keywords:** Computer science, Information technology

## Abstract

Uneven lighting conditions often occur during real-life photography, such as images taken at night that may have both low-light dark areas and high-light overexposed areas. Traditional algorithms for enhancing low-light areas also increase the brightness of overexposed areas, affecting the overall visual effect of the image. Therefore, it is important to achieve differentiated enhancement of low-light and high-light areas. In this paper, we propose a network called correcting uneven illumination network (CUI-Net) with sparse attention transformer and convolutional neural network (CNN) to better extract low-light features by constraining high-light features. Specifically, CUI-Net consists of two main modules: a low-light enhancement module and an auxiliary module. The enhancement module is a hybrid network that combines the advantages of CNN and Transformer network, which can alleviate uneven lighting problems and enhance local details better. The auxiliary module is used to converge the enhancement results of multiple enhancement modules during the training phase, so that only one enhancement module is needed during the testing phase to speed up inference. Furthermore, zero-shot learning is used in this paper to adapt to complex uneven lighting environments without requiring paired or unpaired training data. Finally, to validate the effectiveness of the algorithm, we tested it on multiple datasets of different types, and the algorithm showed stable performance, demonstrating its good robustness. Additionally, by applying this algorithm to practical visual tasks such as object detection, face detection, and semantic segmentation, and comparing it with other state-of-the-art low-light image enhancement algorithms, we have demonstrated its practicality and advantages.

## Introduction

Low-light image enhancement has been studied for many years and has important applications in areas such as night-time video surveillance and autonomous vehicles. Therefore, using low-light image enhancement algorithms to restore low-light images to normal-light images provides a solid foundation for subsequent high-level vision tasks, such as object detection, object tracking, and semantic segmentation. At the same time, low-light image enhancement technology is also indispensable in fields such as military security and deep-sea exploration^1^.

Traditional methods^2–7^ for low-light image enhancement are typically based on histogram equalization and Retinex-based approaches. These methods have some effect in increasing the brightness of low-light images, but they often suffer from over-enhancement and detail loss, as well as excessive noise and color distortion due to the reduction of grayscale levels, scene complexity, and unstable prior knowledge extraction^1^.

With the improvement of computer hardware technology, the speed of data processing has been greatly increased. Many deep learning-based methods^8–13^ have shown good performance in the field of low-light image enhancement. Currently, most low-light image enhancement methods are based on convolutional neural networks (CNNs), which learn the mapping relationship from low-light images to normal-light images from a large amount of data through carefully designed CNN structures. However, the limited receptive field during the convolution operation in CNNs cannot fully consider long-distance pixel relationships in the input image, which affects the image enhancement effect^14^. The self-attention mechanism^15,16^ in Transformers^17–21^ can solve this problem. The self-attention mechanism models long-range dependencies, which can better preserve image details and reduce the impact of noise, thereby improving the quality of the image^22^. Transformer-based methods have made important progress in low-level vision tasks such as image super-resolution^23,24^, image denoising^25^, and image dehazing^26^. Currently, related Transformer methods^27,28^ have also been applied to low-light image enhancement and have achieved good performance, as they can better model non-local information to achieve high-quality image reconstruction. However, these methods do not enhance the local features of the image well, which is what CNNs excel at. Therefore, recent researchr^29–31^ has attempted to combine CNN and Transformer networks to combine their advantages and improve the performance of the corresponding tasks. For low-light enhancement tasks, network architecture design needs to be adapted to the characteristic of low-light images having more low-light features than high-light features. At the same time, for low-light enhancement tasks in real scenes, zero-shot learning^32^ methods are needed to better solve high-level vision tasks in real scenes where paired datasets are lacking. Specifically, zero-shot learning means that no paired or unpaired data is needed during training.

The substantial contributions of this study are meticulously designed to combat the issue of uneven illumination. Transformers, armed with their global attention mechanism, can comprehensively process long-range pixel relations in an input image. However, the traditional self-attention mechanism demands a high quantity of computational resources, and its multitude of parameters could lead to overfitting. On the other hand, CNN networks are well-regarded for enhancing local features and maintaining robustness. Still, they struggle in capturing global context information. The integration of these two networks, without thoughtful design of the CNN network, could lead to an ineffective learning of global information features generated by the Transformer network.

Aiming to unite the advantages of CNN’s local feature extraction and Transformer’s global modeling, the network introduced in this study comes with specific improvements. The complexity of the Transformer module increases linearly, not quadratically, with the rise in image resolution, facilitating efficient acquisition of contextual information. The CNN module’s class Transformer structure is designed to concentrate better on the features extracted by the Transformer, making up for the difficulties in global information acquisition and thus enhancing the model’s efficiency^33^. Ablation experiments were conducted during the development process, and multiple combinations were tested before finalizing the network architecture presented in this paper.

Particularly, the channel attention mechanism of the auxiliary module and the Multi-Dconv head Sparse Attention(MDSA) module designed in this research addresses to some extent the issue of high time and space complexity inherent to traditional Transformers. The introduction of the sparse attention mechanism provides a deeper understanding and handling of the local features in the image. In low-light enhancement tasks, overly bright local features may hinder the model’s ability to capture other critical low-light features. To mitigate this problem, the MDSA module is adopted for a more precise depiction of local features and to boost their enhancement ability, marking the first application of the improved sparse attention mechanism in low-light enhancement tasks.

**Figure 1:**
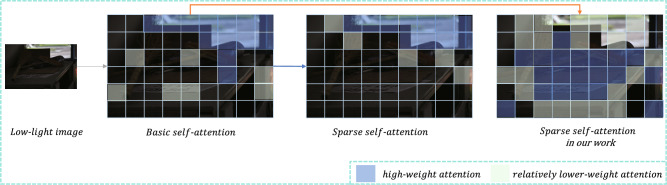
It depicts the handling strategies of different attention mechanisms under the conditions of unevenly lit low-light environments. The traditional self-attention mechanism generally prefers to place its main focus on highlight features. Furthermore, the conventional sparse self-attention mechanism tends to concentrate a significant portion of the weights on the highlight features. Such an approach is not ideal for low-light enhancement tasks because it results in a tendency for overexposure in highlight areas while inhibiting sufficient enhancement of details in low-light areas. However, our proposed sparse self-attention mechanism breaks away from this norm. It is capable of appropriately shifting the majority of the weights towards low-light features while simultaneously effectively reducing the weights of highlight features. This facilitates a more balanced extraction and processing of features.

Figure 1 illustrates that in unevenly lit low-light environments, conventional self-attention mechanisms or ordinary sparse self-attention mechanisms tend to place the primary focus and weight on the highlight features, which is not ideal for low-light enhancement tasks. The sparse self-attention mechanism applied in this study properly biases the main weight towards low-light features while effectively reducing the weight of highlight features, significantly improving the model’s performance in low-light enhancement tasks. This method, unexplored in original methodologies, represents innovative thinking.

Among the two inputs in the Cross Gating Feedforward Network(CGFN) module, one is processed through the MDSA module, and the other bypasses it. The MDSA module implements the sparse attention mechanism on the channel dimension. Therefore, the proposed CGFN calculates weights in the spatial dimension, addressing the lack of spatial information after the feature passes through the MDSA module. Additionally, the presence of the gating mechanism can better suppress the further propagation of information features that are unfavorable to model convergence. In low-light enhancement tasks, the feature information in the highlight area can severely hamper the enhancement quality. The CGFN module can further alleviate this problem, introducing a method not previously seen in other methodologies.

Therefore, considering the characteristics of low-light images under uneven lighting, this article proposes a more effective zero-shot learning low-light enhancement network structure. The main contributions are summarized as follows:

- A zero-shot learning low-light enhancement network named CUI-Net was designed. The entire network comprises enhancement modules and auxiliary modules. The enhancement module merges the global attention mechanism of the Transformer and the ability of the CNN network to process local features. It has efficient computing efficiency and powerful modeling capabilities. This unique structure enables better handling of the problem of uneven lighting, richer feature information extraction, and achievement of image enhancement in low-light environments. The CNN network in the auxiliary module augments the convergence ability of the enhancement module and indirectly rectifies the influence of lighting.
- A Multi-Dconv head Sparse Attention (MDSA) module was designed. The MDSA module constrains highlight features at the channel level and increases the weight of important local features. This design helps quell the interference of overly bright features, allowing the model to focus on and extract low-light features better, thereby enhancing the model’s performance in low-light enhancement tasks.
- A novel Cross Gating Feedforward Network (CGFN) was proposed. CGFN can not only effectively suppress the further spread of information features that are not conducive to model convergence but also supplement the information loss in the spatial dimension through information exchange, thereby further boosting the efficiency and effect of the model. For low-light enhancement tasks, the feature information in the highlight area can seriously disrupt the enhancement quality of low-light enhancement tasks. The existence of the CGFN module can further mitigate this problem.
- A multitude of experiments was conducted on nine challenging datasets. Most of the experimental results indicate that CUI-Net surpasses current state-of-the-art methods in terms of image quality enhancement effects and various evaluation indicators. More importantly, CUI-Net’s superior performance in high-level visual tasks (such as object detection, face detection, and semantic segmentation) in real-world low-light scenarios further validates its practical value and effectiveness.

## Related work

### Traditional enhancement methods

Traditional low-light enhancement methods can be primarily divided into two types: the methods based on histogram equalization (HE) and the methods based on the Retinex model. Methods based on HE^2,3^ redistribute pixel values based on the cumulative distribution function of the input image to expand the dynamic range. However, these methods are also prone to color fidelity loss and the generation of noise, resulting in image distortion^4^. The Retinex theory^5^ decomposes low-light images into reflectance part and illumination part based on prior knowledge or regularization, such as the Single Scale Retinex model (SSR)^6^ and the Multi-Scale Retinex model (MSR)^7^. MSR is considered a weighted sum of several different SSR outputs. The output of these methods may cause changes. The relative proportions of the enhanced three color channels can be affected. Compared to the original image, this can lead to color distortion^4^. Fu et al.^34^ proposed a fusion method that combines the advantages of sigmoid function and histogram equalization, which has improved performance compared to^2,3^. Guo et al.^35^ initialized the illumination map of the image by finding the maximum value in the RGB channels and then optimized the initial illumination map by adding a structural prior to achieve image enhancement. These methods have some effect in increasing the brightness of low-light images, however, some algorithms ignore the correlation between the bright and dark parts, resulting in color distortion in images with significant brightness differences.

### Deep learning-based methods

Most of the network-based low-light image enhancement algorithms are based on CNN methods. In CNN-based methods, methods based on Retinex usually enhance the illumination and reflection components separately through dedicated sub-networks^32^. Wei et al.^36^ introduced the Retinex-Net model, which aims to enhance low-light images. The model comprises two parts: a Decom-Net for decomposing images into illumination and reflection components, and an Enhance-Net for adjusting the illumination. Despite its purpose, Retinex-Net unfortunately results in significant color distortion, leading to less natural-looking enhanced images^8^. EnlightenGAN^9^ uses a Generative Adversarial Network (GAN) ,which used U-Net^10^ based on attention mechanisms as generator and a global-local discriminator to obtain the enhancement results. ZeroDCE^11^ trains a lightweight network (DCE-NET) to fit the brightness mapping curve, and it is then used to adjust the brightness distribution of the image. Retinex- inspired Unrolling with Architecture Search (RUAS)^12^ uses an unfolding architecture search to handle low-light image enhancement. Self-Calibrated Illumination (SCI)^13^ proposes a simplified network that fits physical principles to achieve low-light enhancement and introduces a calibration process in the training stage to improve the low-light enhancement model’s ability, thereby further improving the enhancement effect.

### Methods combining CNN and transformer

CNN operations provide efficiency and universality, but their receptive fields are limited and cannot fully consider long-range pixel relationships in input images, which can affect image enhancement performance. In contrast, in Transformers, the self-attention mechanism focuses on modeling long-range dependencies, enabling it to capture global information well. However, it lacks attention on the most relevant information^37^ and its complexity grows exponentially with spatial resolution^14^, leading to poor performance in some tasks. Thus, combining the two effectively to improve image enhancement quality is the focus of this paper. Conformer^29^ uses a CNN branch and a Transformer branch and combines them through Feature Coupling Units to fuse local convolution blocks, self-attention modules, and MLP units to adjust feature resolution and channel numbers while continually eliminating semantic differences between the CNN and Transformer branches. HNCT^30^ integrates CNN and Transformer while using local and non-local priors to extract features beneficial for super-resolution and an enhanced spatial attention module to further improve performance. ECFAN^31^ proposes a new hybrid super-resolution method, called ACT, that combines CNN and Vision Transformer^19^ to effectively aggregate local and non-local features and introduces cross-scale token attention modules to effectively utilize multi-scale token representations.

Through careful consideration and experimental comparison, we have found that our method uses three TransformerBlocks as the encoder to preserve the most useful self-attention values, avoiding the further propagation of aggregated highlight features, allowing useful global features to be fully utilized, and transmitting useful local features to ensure that the enhanced low-light images have sufficient details. Two CNN blocks serve as the decoder to further utilize the feature information obtained from the Transformer blocks to better enhance the details and texture information of low-light images, leveraging the advantages of CNN networks.

### Sparse attention

Images captured in real-world scenarios often suffer from uneven illumination^32^. For example, images taken at night may contain both dark and bright areas or overexposed regions, such as areas around light sources. Existing methods often enhance both the dark and bright regions of the image simultaneously, which can affect the visual quality of the enhancement results. However, current low-light image enhancement methods have not fully addressed this open problem. Zhao et al.^38^ proposed sparse Transformer to select the attention degree of the model. Fu et al.^37^ proposed a target focus network and sparse Transformer technique for visual object tracking. The target focus network focuses on the target of interest in the search region and highlights the features of the most relevant information for better estimating the states of the target. Inspired by SparseTT^37^, we adapt sparse Transformer to the low-light enhancement task. For low-light images with uneven illumination, the Transformer is susceptible to the influence of high-light features when computing self-attention, resulting in higher attention values. This naturally leads to a bias towards enhancing high-light features rather than low-light features with low attention values when modeling global feature dependencies. Therefore, we propose a sparse attention operation that differs from the usual one, choosing to set high-light features to lower values to effectively suppress high-light information and focus on the most relevant information in low-light enhancement tasks.

## Proposed method

In this section, the framework of CUI-Net and the two main modules: which is the enhancement module will be introduced and the auxiliary module. Finally, we will explain the unsupervised training losses used in our neural network model.

### Overall procedure

**Figure 2:**
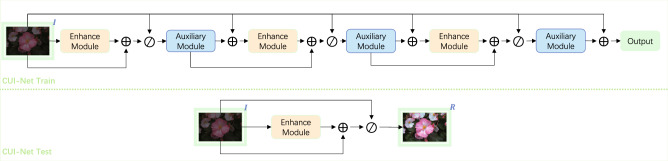
Overall framework of the CUI-Net. Only one enhancement module is used to obtain results during the testing phase.

The proposed CUI-Net is a cascaded two-stage image enhancement network (Fig. 2) In the first stage, a Transformer network is introduced to obtain global information, which can better enhance the details of low-light images. In the second stage, an auxiliary network based on multiple convolutional network blocks is constructed, and the original input image is used as a constraint to control the output detail features of the first stage. Unlike traditional methods, the training part of CUI-Net requires multiple Enhancement Modules and Auxiliary Modules, while the testing part only contains Enhancement Module.

Here, assume that the low-light input image $I\in {\mathbb {R}}^{H\times W\times C}$. where the height is *H*, the width is *W*, and the number of channels is *C*. For *RGB* images, *C* is equal to 3. According to the Retinex theory, the low-light image *I* can be obtained by performing the following operation on the clear image *R* and the illumination image *L*^5^:

1 $$\begin{aligned} I = R\otimes L \end{aligned}$$

Therefore, the enhanced image *R* can be obtained through input image *I* and the illumination map *L*.

During the training process, the entire framework can be divided into two parts, namely the Enhancement Module (EM) and the Auxiliary Module (AM):

2 $$\begin{aligned} \varepsilon _{t}= & {} E\!M_{t}({\mathscr {A}}_{t-1}+I;\vartheta )+{\mathscr {A}}_{t-1 } \end{aligned}$$

3 $$\begin{aligned} {\mathscr {A}}_{t}= & {} A\!M_{t}(I\oslash \varepsilon _t;\mu ),\gamma _{0}=I \end{aligned}$$

where $EM_{t}$ is the $t$-th image enhancement module network with learnable parameters $\vartheta$, and $AM_t$ is the $t$-th auxiliary module network with learnable parameters $\mu$. When $t=1$, i.e., in $EM_1$, only the original low-light image *I* is used as input, i.e. $EM_1 (I;\vartheta )$, and the original low-light image *I* is not added as input.

Unlike the training part, the auxiliary module is not needed in the testing part, and only one enhancement module is used to obtain the clear image.

4 $$\begin{aligned} R = I\oslash [EM(I;\vartheta )+I] \end{aligned}$$

### Image enhancement module

The image enhancement module consists of an efficient Transformer block and a CNN block, serving as the encoder and decoder, respectively. The Transformer model enhances low-light images by filtering out information from uneven lighting channels and local details, and then transferring the useful features to the next part of the network. The core of the Transformer block lies in the Multi-Dimensional Sparse Attention (MDSA) mechanism and the Cross-Gated Feed-Forward Network (CGFN). MDSA can effectively reduce redundant features and improve the weights of important features, thus enhancing the network’s robustness and generalization ability. The cross-gated mechanism can compensate for the lack of information in the spatial dimension, allowing useful information to propagate further and enhance the integrity of the entire feature representation. The CNN block replaces the attention block in the traditional Transformer network with deep convolutions and the feed-forward layer with a simplified CNN structure, ensuring lightness. Meanwhile, a structure similar to the Transformer network can further process feature information and has the generality and efficiency advantages of a convolutional neural network.

In summary, the channel-wise sparse attention and cross-gated Transformer are used as the encoder in the image enhancement module. With the increase of layers number, the extracted features become increasingly abstract and semantically rich. The CNN block is used as the decoder to extract and enhance features at a higher level, making it more suitable for image enhancement tasks in uneven lighting conditions. Realizing pixel-level information transfer and context association through convolution calculation can further improve the performance and efficiency of the model.

**Figure 3:**
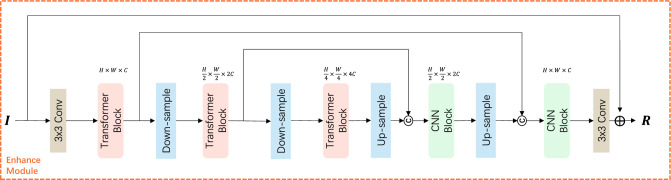
Network architecture of the enhancement module.

**Figure 4:**
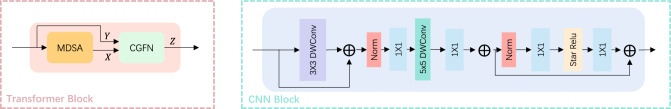
The network structure diagrams of the Transformer module used in the enhancement module and the CNN module used in both the enhancement and auxiliary modules.

The specific process of the image enhancement module is shown in Fig. 3. The network structure diagrams of the Transformer and CNN modules in the enhancement module are shown in Fig. 4. First, the input low-light image *I* undergoes a $3\times 3$ convolutional operation to extract low-level features and increase the number of channels. It then passes through three Transformer encoders and two CNN decoders. The residual connections and upsampling and downsampling operations are utilized to extract sufficient detail features. Finally, a $3\times 3$ convolutional operation is used to restore the original number of channels, and the resulting image is added to the input low-light image *I* to produce the final output image. *C* stands for Concatenation operation.

### Multi-Dconv head sparse attention

In traditional Transformer modules, multi-head self-attention mechanisms compute global information through self-attention mechanisms in the spatial dimension, resulting in a quadratic growth in complexity with increasing resolution. The main purpose of sparse attention mechanisms is to reduce the time and space complexity of traditional Transformers^39^. In this paper, the channel attention mechanism used in the MDSA module not only reduces model complexity and improves efficiency, but also helps the model better understand local features in the image. In low-light enhancement tasks, the appearance of too many high-brightness local features may interfere with the model’s ability to capture other low-light features. Therefore, this paper uses sparse attention mechanisms to assist the model in better representing local features and improving its enhancement ability.

**Figure 5:**
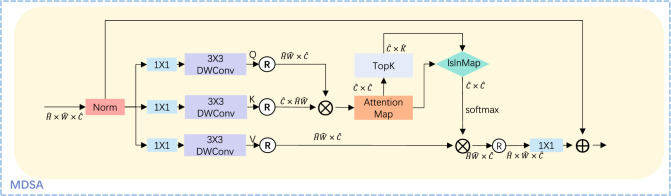
Network structure diagram of MDSA module.

The specific structure of MDSA is shown in Fig. 5. The input tensor is denoted as $I\in {\mathbb {R}}^{{\hat{H}}\times {\hat{W}}\times 3}$. *Q*, *K*, and *V* represent query, key, and value. The $1\times 1$ point-wise convolution is applied to aggregate pixel-level cross-channel context, followed by a $3\times 3$ depth-wise convolution to encode channel-level spatial context. The operation $\circledR$ in the figure stands for reshape. $I\!s\!I\!n\!M\!ap$ is used to filter out the weights in the attention map matrix that are the same as the weights in the TopK matrix, and set the corresponding weights in the attention map to 0.01.

Different from the Vision Transformer model^19^, MDSA uses self-attention mechanism to calculate the similarity between each channel, i.e., attention calculation is performed on the channel dimension rather than on the spatial dimension. This enables MDSA to better capture the relationships between feature channels, thereby improving the model’s representation ability and robustness.

Specifically, the TopK operation is performed on the attention map to select the top *K* attention values, followed by further operations. It should be noted that, unlike general sparse attention calculation, in the low-light task under uneven illumination, the channel information of the high-light area in the attention map is more likely to receive higher attention scores. These *K* attentions need to be set to 0.01 to allow the low-light channel features to be sent to CGFN for obtaining the required local information.

5 $$\begin{aligned} {\hat{X}}= & {} W_pSpAttention({\hat{Q}},{\hat{K}},{\hat{V}})+I \end{aligned}$$

6 $$\begin{aligned} SpAttention({\hat{Q}},{\hat{K}},{\hat{V}})= & {} {\hat{V}}\cdot softmax(TopK({\hat{K}}\cdot {\hat{Q}}/\lambda )) \end{aligned}$$

Here, ${\hat{Q}}\in {\mathbb {R}}^{{\hat{H}}{\hat{W}}\times {\hat{C}}},{\hat{K}}\in {\mathbb {R}} ^{{\hat{C}} \times {\hat{H}}{\hat{W}}},{\hat{V}}\in {\mathbb {R}} ^{{\hat{H}}{\hat{W}}\times {\hat{C}}}$ are obtained by reshaping the original scale ${\mathbb {R}}^{{\hat{H}}\times {\hat{W}}\times {\hat{C}}}$. The meaning of *SpAttention* is sparse attention. $W_p$ represents a $1\times 1$ point-wise convolution. $\lambda$ is a learnable scaling parameter used to control the magnitude of the dot product of ${\hat{K}}$ and ${\hat{Q}}$.

### Cross-gated feed-forward network

The two inputs of the Cross-Gated feed-forward Network (CGFN) are the input and output obtained through MDSA. The cross-gating part is equivalent to calculating weights on the spatial dimension and weighting specific positions, in order to compensate for the lack of spatial dimension information in the image that has not passed through MDSA.

**Figure 6:**
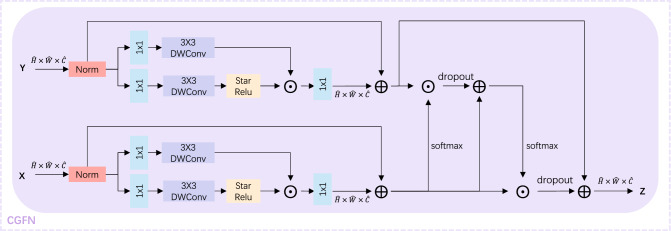
Network structure diagram of CGFN module.

The specific structure of the CGFN is shown in Fig. 6. Each single path of the CGFN module has two branches. One branch is a gating unit used to obtain the activation state of each pixel. The $1\times 1$ convolutional layer is used to expand the channel number, followed by a $3\times 3$ depthwise convolutional layer and StarReLU to generate the gate map. The other branch does not need to pass through the StarReLU activation function. Then, the two branches are dot-multiplied. The cross-gating is cross-calculated on the two paths to compensate for the lack of spatial information. If the input of CGFN from MDSA is $X\in {\mathbb {R}}^{{\hat{H}}\times {\hat{W}}\times {\hat{C}}}$ , $Y\in {\mathbb {R}}^{{\hat{H}}\times {\hat{W}}\times {\hat{C}}}$ is the input from the previous module without MSDA then the CGFN can be represented as follows:

7 $$\begin{aligned} {\hat{Z}}= & {} W_o^{2}((W_m^{2}(W_o^{1}(W_m^{1}({\hat{X}})\odot {\hat{Y}})+{\hat{X}}))\odot {\hat{X}}) + {\hat{Y}} \end{aligned}$$

8 $$\begin{aligned} {\hat{X}}= & {} W_p^0G(X)+X \end{aligned}$$

9 $$\begin{aligned} {\hat{Y}}= & {} W_p^0G(Y)+Y \end{aligned}$$

10 $$\begin{aligned} G(X)= & {} \phi (W_d^1 W_p^1(LN(X)))\odot W_d^2 W_p^2 (LN(X)) \end{aligned}$$

where $\odot$ denotes element-wise multiplication, $\phi$ represents the StarReLU non-linear activation function, and *LN* stands for Layer Normalization. $W_m$ performs softmax operation. $W_o$ performs dropout operation. ${\hat{Z}}$ will serve as the input to the next module.

### Auxiliary module

The auxiliary module is necessary for unsupervised image enhancement methods as they may have limitations such as over-enhancement and color bias^8^. Therefore, the CNN network with high efficiency and generalization ability is chosen as the auxiliary module to converge the outputs of multiple enhancement modules to one enhancement effect, enabling the use of one enhancement module during the testing phase to achieve the same enhancement effect as the multiple enhancement modules during the training part.

As shown in Fig. 2 , Formulas (2) and (3), the purpose of the auxiliary module is to correct the input of the enhancement module, indirectly affecting the output of the enhancement module. The input of the auxiliary module can be obtained by element-wise addition of the output of the previous enhancement module and the output of the auxiliary module, followed by division with the original low-light image. Thus, the auxiliary module can obtain the features of the enhancement module and correct the uneven illumination through the original low-light image.

**Figure 7:**
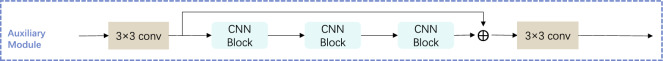
Overall architecture diagram of the auxiliary module.

The auxiliary module uses depth-wise convolution multiple times, which can effectively reduce the number of parameters and computation cost, as shown in Fig. 7. Firstly, the input image is passed through a $3\times 3$ convolution layer to increase the channel number, and then through three CNN blocks. Finally, a $3\times 3$ convolution layer is used to reduce the channel dimension. As shown in Fig. 4, the CNN block enhances the local details by passing the input features through depth-wise convolutions of $3\times 3$ and $5\times 5$, followed by StarReLU activation function and multiple $1\times 1$ convolutions to minimize the number of parameters. The corrected illumination information is then inputted to the enhancement module, improving the enhancement effect of the enhancement module.

### Training loss

In order to consider color preservation, artifact removal and gradient backpropagation, the loss function needs to be optimized. The loss function used by CUI-Net is as follows:

11 $$\begin{aligned} {\mathscr {L}} = \alpha {\mathscr {L}}_c + \beta {\mathscr {L}}_s \end{aligned}$$

Here, *L* represents the total loss, ${\mathscr {L}}_c$ and ${\mathscr {L}}_c$ represent the correction loss and the smoothness loss respectively, and $\alpha$ and $\beta$ are two positive balancing parameters. In the experiments, the balancing parameters are set to $\alpha =1.5$ and $\beta =1$. The correction loss ${\mathscr {L}}_c$ is to ensure the consistency between the estimated illumination and the adjusted result, that is:

12 $$\begin{aligned} {\mathscr {L}}_c = \sum _{x = 1}^{3} {\parallel EM_x - AM_{x-1} \parallel }^2 \end{aligned}$$

Here, $EM_x$ is the x-th enhancement module, and $AM_x$ is the x-th auxiliary module. $AM_0$ is the original input *I*. As an unsupervised loss, this loss function only constrains the output through the auxiliary module.

Then, the smoothness loss is used^40^, that is:

13 $$\begin{aligned} {\mathscr {L}} _s = \sum _{i = 1}^{N} \sum _{j\in {\mathscr {N}}(i)}^{} Weight_{i,j}\mid X_i^t - X_j^t\mid \end{aligned}$$

Here, *N* is the total number of pixels. *i* is the *i*-th pixel. ${\mathscr {N}}(i)$ represents the neighboring pixels in its 5 × 5 window. $Weight_{i,j}$ represents the weight, which is specified as equation 14, where *c* represents the image channel in the YUV color space, and $\sigma =0.1$ is the standard deviation of the Gaussian kernel.

14 $$\begin{aligned} Weight_{i,j} = exp(-\frac{\sum _{c}{((y_{i,c}+s_{i,c}^{t-1})-(y_{j,c}+s_{j,c}^{t-1}))}^2}{2\sigma ^2}) \end{aligned}$$

## Experiment

To test the effectiveness of the algorithm, this paper verifies it on multiple datasets and tasks. Firstly, the experimental settings are given, and tests are conducted on public datasets to demonstrate the effectiveness of the algorithm through quantitative comparison and qualitative analysis with existing methods. Then, high-level tasks, including low-light object detection, dark face detection, and nighttime semantic segmentation, are tested and compared with existing algorithms to further validate the effectiveness of the algorithm. Finally, ablation experiments are conducted to verify the effectiveness of each module.

### Experimental settings

The experiment is based on PyTorch and conducted on a computer with an Intel i9-10940X CPU, two RTX 3090 GPUs, and 32GB of memory for training and testing. The main parameters are batch size of 1, initial learning rate of $10^{-4}$, weight decay of $\epsilon =10^{-8}$, and training epoch of 500. In the enhancement module, the number of Transformer blocks is set to 4, 6, 6, and 8 from the first layer to the fourth layer, the number of attention heads in MDTA is set to 2, 4, and 8, and the number of channels is set to 48, 96, and 192. StarReLU^41^ and Adan^42^ optimizer are introduced in CUI-Net. StarReLU is a variant of Squared ReLU designed to eliminate distribution shift. StarReLU performs well in both algorithm performance and computational efficiency due to reducing the computational cost of the activation function^43^. Adan can complete the training of ViT^19^ with only half the computational cost. Compared with the popular optimizer Adam^44^, Adan has an additional hyperparameter $\beta _2$ for adjustment. $\beta _2$ is set to 0.08 in the experiments^42^.

Here, $EM_x$ represents the x-th enhancement module, and $AM_x$ represents the x-th auxiliary module. $AM_0$ represents the original input *I*. As an unsupervised loss, the loss function *L* only constrains the output through the auxiliary module.

To verify the effectiveness and superiority of the proposed algorithm, CUI-Net is compared with state-of-the-art (SOTA) methods, including EnlightenGAN^9^, KinD^45^ , ZeroDCE^11^, ZeroDCE++^46^, RUAS^12^, SCI^13^, and Uretinex-Net^47^. Additionally, comparisons are made in high-level vision tasks such as face detection, object detection, and semantic segmentation.

### Benchmark description and evaluation metrics

For image enhancement testing, 100 random images from the MIT dataset^48^ and 50 random images from the LSRW dataset^49^ are used for testing. To quantitatively measure the algorithm’s performance, three full-reference metrics, including PSNR, SSIM, and LPIPS^50^, and four no-reference metrics, including NIQE^51^, ILNIQE^52^, NIMA^53^, and MUSIQ^54^, are used as evaluation metrics.

For dark face detection tasks, the DARK FACE dataset^55^, consisting of 1000 challenging test images, is used. 500 random images are selected as the training set, and 50 images are used for testing, with the average precision (AP) used as the evaluation metric.

For low-light object detection tasks, the ExDark dataset^56^ specifically designed for low-light object detection is used. 1051 images are selected as the training set, and 406 images are used for testing, with evaluation metrics including $mAP_{0.5:0.95}$ and $mAP_{0.5}$.

For nighttime semantic segmentation tasks, the ACDC dataset^57^ is used. The ACDC dataset is a self-driving dataset released in ICCV 2021. 400 dark condition images are used for training, and the remaining 106 images are used as the test set. The evaluation metrics include IoU and mIoU.

### Quantitative and qualitative metrics

The quantitative results on the MIT dataset are shown in Table 1. CUI-Net achieved the best performance in SSIM, PSNR, LPIPS, and ILNIQE among the seven evaluation metrics. Specifically, CUI-Net achieved a PSNR of 193.328dB, which is 1.0259dB higher than the best existing best algorithm’s score of 18.3201dB, and an ILNIQE evaluation metric has a score of 31.9151, which is 1.5756 lower than the score of the best existing algorithm.

**Table 1:** Quantitative results of three supervised metrics (PSNR, SSIM, and LPIPS) and four no-reference metrics (NIQE, NIMA, MUSIQ, and ILNIQE) on the MIT dataset.

Method	SSIM$\uparrow$	PSNR$\uparrow$	LPIPS$\downarrow$	NIQE$\downarrow$	NIMA$\uparrow$	MUSIQ$\uparrow$	ILNIQE$\downarrow$
EnlightGAN	0.7979	16.5871	0.204	**4.1572**	3.3962	55.4027	34.6982
KinD	0.8037	16.8844	0.1982	4.2655	*3.8035*	49.2749	**33.4907**
ZeroDCE	0.7755	16.0836	0.2261	4.8881	3.2382	**58.8717**	37.6914
ZeroDCE++	0.4356	6.6687	0.5018	5.5502	3.3837	47.0083	36.7629
RUAS	0.8301	**18.3021**	0.2086	4.7324	3.5027	54.7674	35.9107
SCI	*0.8354*	18.42	**0.1752**	4.4377	3.4981	57.8329	34.0613
Uretinex	0.7694	16.166	0.2135	4.5749	**3.6984**	*60.7205*	34.9065
CUI	**0.8305**	*19.328*	*0.1736*	*4.1532*	3.4726	56.9714	*31.9151*

The best and second best results are highlighted in italic and bold, respectively.

**Figure 8:**
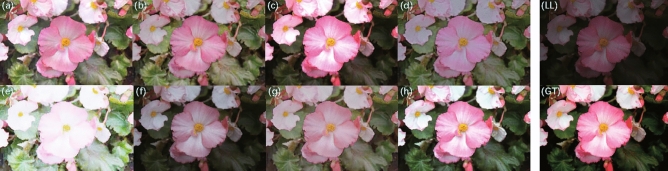
Enhancement results on the MIT dataset: (**a**) EnlightenGAN; (**b**) kinD; (**c**) RUAS; (**d**) ZeroDCE; (**e**) ZeroDCE++; (**f**) SCI; (**g**) Uretinex; (**h**) CUI-Net; (LL) is the input low-light original image; (GT) is the ground truth with sequence number E.

The enhancement results on the MIT dataset are shown in Fig. 8. Compared with the ground truth (Fig. 8GT) for the input low-light original image (Fig. 8LL), EnlightenGAN (Fig. 8a), KinD (Fig. 8b), ZeroDCE (Fig. 8d), SCI (Fig. 8f), and Uretinex (Fig. 8g) methods show inadequate enhancement, while ZeroDCE++ (Fig. 8e) shows over-enhancement. RUAS (Fig. 8c) enhances the white petals on the upper part of the image into pinkish color, but the overall saturation is too high. In contrast, CUI-Net Fig. 8h) shows better color restoration while maintaining realistic lighting conditions.

The quantitative results on the LSRW dataset are shown in Table 2 . Among the seven evaluation metrics, CUI-Net achieved the best result in the NIMA and the third-best results in the PSNR, NIQE and MUSIQ. Uretinex achieved good results on the LSRW dataset, which may be because the data augmentation method of the LSRW dataset is similar to that of the LOL dataset used in supervised training of Uretinex. However, our unsupervised method may be less sensitive to artificially augmented datasets.

**Table 2:** Quantitative results of three supervised metrics (PSNR, SSIM, and LPIPS) and four non-reference metrics (NIQE, NIMA, MUSIQ, and ILNIQE) on the LSRW dataset.

Method	SSIM$\uparrow$	PSNR$\uparrow$	LPIPS$\downarrow$	NIQE$\downarrow$	NIMA$\uparrow$	MUSIQ$\uparrow$	ILNIQE$\downarrow$
EnlightGAN	***0.5032***	**17.08**	0.3274	*3.4691*	4.2444	56.3958	***27.6677***
KinD	**0.5039**	16.4069	0.3371	**3.5432**	**4.5964**	**61.2283**	*27.231*
ZeroDCE	0.4773	15.8545	**0.3172**	3.7209	3.9323	55.8506	30.806
ZeroDCE++	0.4229	12.9252	0.4324	3.7084	3.887	50.335	**27.6368**
RUAS	0.4324	14.0305	0.3846	4.2598	4.2804	53.9967	36.2296
SCI	0.4474	15.2405	***0.322***	3.8659	4.276	56.2192	32.1176
Uretinex	*0.5453*	*18.2709*	*0.2994*	4.1814	***4.5923***	*65.8647*	29.642
CUI	0.4389	***16.564***	0.333	***3.6239***	*4.6053*	***56.6684***	29.6124

The best result is highlighted in italic, the second-best is highlighted in bold, and the third-best is highlighted in bolditalic.

**Figure 9:**
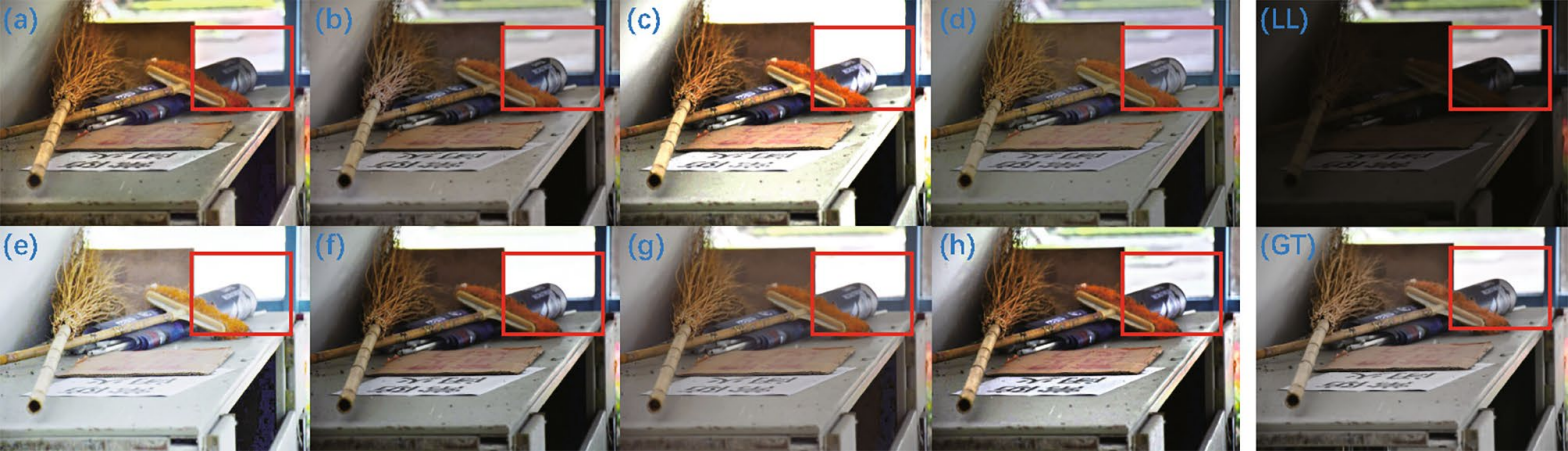
Enhanced images on the LSRW dataset: (**a**) EnlightenGAN; (**b**) kinD; (**c**) RUAS; (**d**) ZeroDCE; (**e**) ZeroDCE++; (**f**) SCI; (**g**) Uretinex; (**h**) CUI-Net; (LL) the input low-light image; (GT) the ground truth.

**Figure 10:**
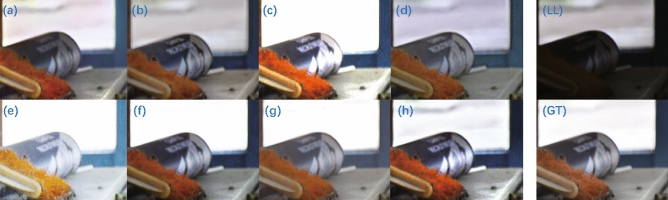
Details of the corresponding enlarged areas in Fig. 9 of the LSRW dataset: (**a**) EnlightenGAN; (**b**) kinD; (**c**) RUAS; (**d**) ZeroDCE; (**e**) ZeroDCE++; (**f**) SCI; (**g**) Uretinex; (**h**) CUI-Net; (LL) the input low-light image; (GT) the ground truth.

The enhancement results on the LSRW dataset are shown in Fig. 9. Except for ZeroDCE++ (Fig. 9e), which shows over-enhancement, the overall enhancement effect of the EnlightenGAN (Fig. 9a), KinD (Fig. 9b), RUAS (Fig. 9c), ZeroDCE (Fig. 9d), ZeroDCE++ (Fig. 9e), SCI (Fig. 9f), Uretinex (Fig. 9g), and CUI-Net (Fig. 9h) methods is similar. By enlarging the selected local areas for detailed comparison, we observed two parts of the scene: the outdoor and indoor scenes are observed separately. RUAS (Fig. 10c), ZeroDCE++ (Fig. 10e), and SCI (Fig. 10f) showed over-exposure in the outdoor scenes. Uretinex (Fig. 10g), which achieved better quantitative results, also showed over-exposure. It is worth noting that even the ground truth (Fig. 10GT) shows over-enhancement in the outdoor scenes compared to the low-light original image (Fig. 10LL). Since CUI-Net (Fig. 10h) can suppress highlight areas under uneven lighting conditions, better enhancement of outdoor scenes may not always contribute to some evaluation metrics. For indoor scenes, EnlightenGAN (Fig. 10a), KinD (Fig. 10b), and ZeroDCE (Fig. 10d) resulted in blurred text and less realistic surface reflections, while CUI-Net can not only enhance the details and contours of low-light areas but also restore the realistic lighting conditions of the scene. In addition, CUI-Net can enhance the text on the white paper and paper box on the desk more clearly, which may have practical applications in low-light image text extraction tasks.

Although CUI-Net has some shortcomings in quantitative metrics on the LSRW dataset, the qualitative analysis of the enhancement results shows some discrepancies between the relevant metrics and subjective observations in practical applications.

**Figure 11:**
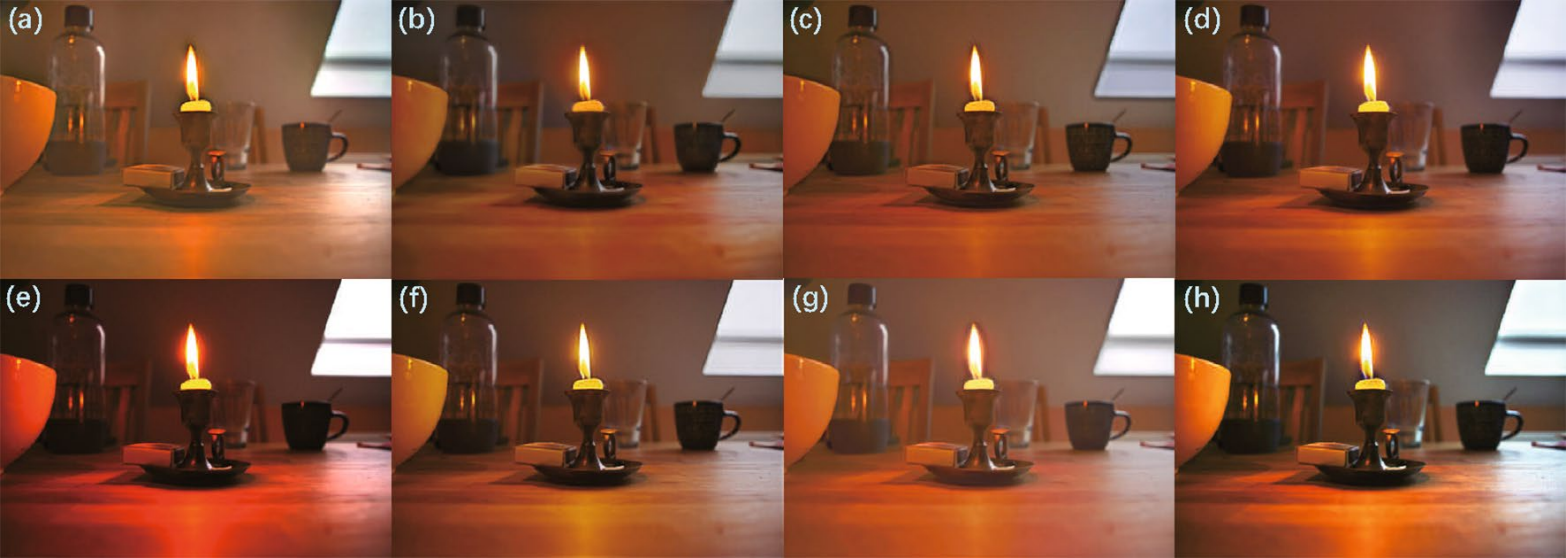
Test result display on the MEF dataset: (**a**) EnlightenGAN; (**b**) kinD; (**c**) RUAS; (**d**) ZeroDCE; (**e**) ZeroDCE++; (**f**) SCI; (**g**) Uretinex; (**h**) CUI-Net.

**Figure 12:**
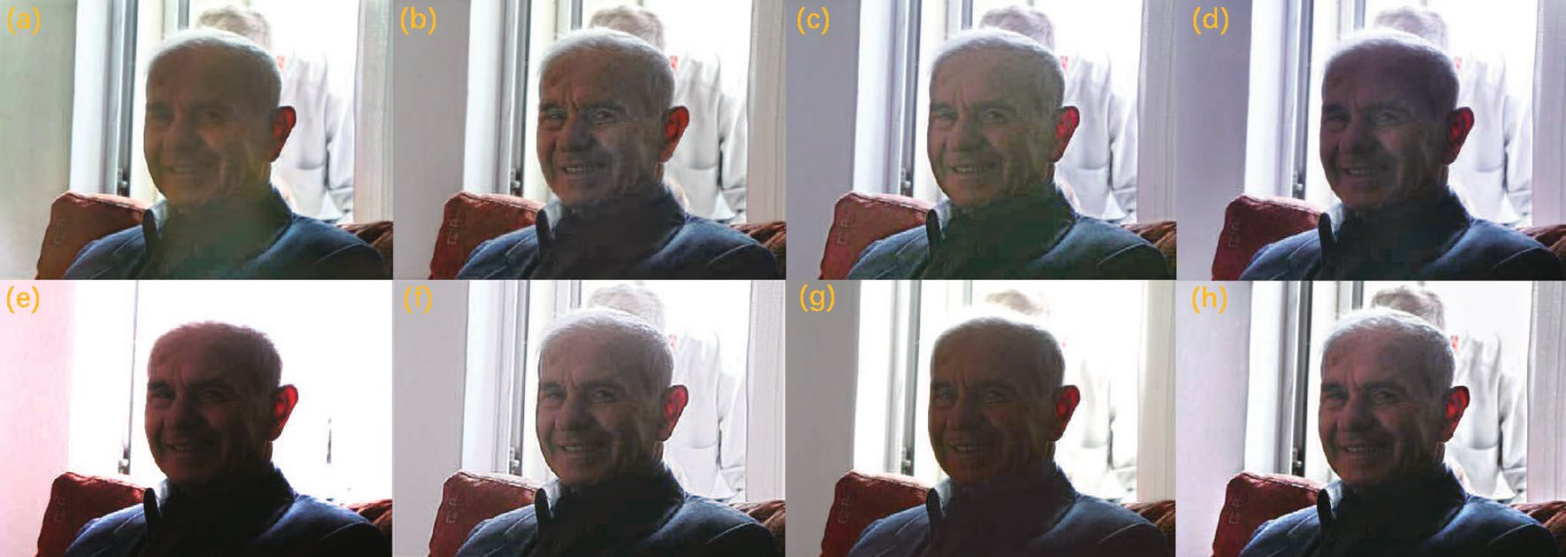
Test result display on the VV dataset: (**a**) EnlightenGAN; (**b**) kinD; (**c**) RUAS; (**d**) ZeroDCE; (**e**) ZeroDCE++; (**f**) SCI; (**g**) Uretinex; (**h**) CUI-Net.

**Figure 13:**
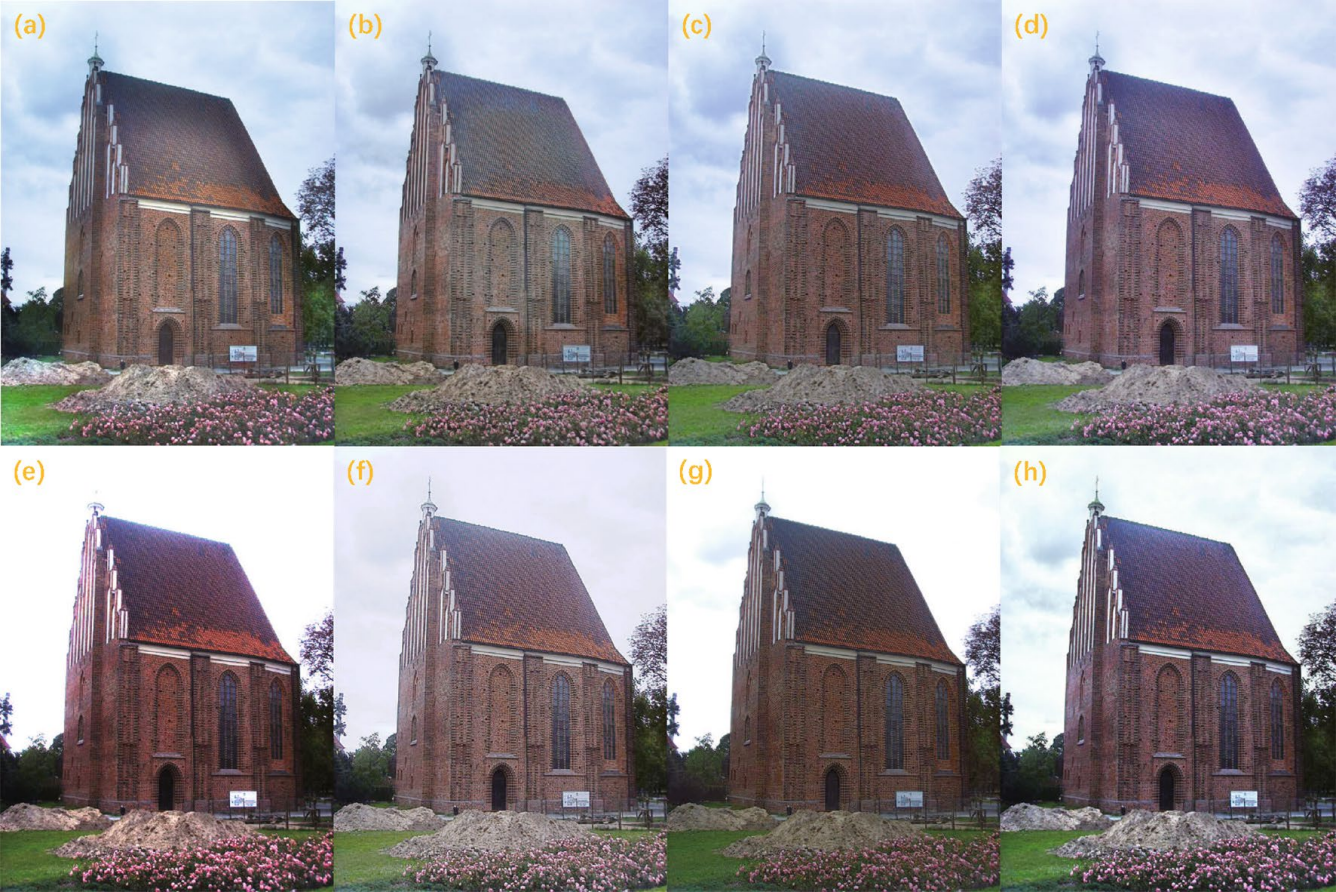
Test result display on the DICM dataset: (**a**) EnlightenGAN; (**b**) kinD; (**c**) RUAS; (**d**) ZeroDCE; (**e**) ZeroDCE++; (**f**) SCI; (**g**) Uretinex; (**h**) CUI-Net.

**Figure 14:**
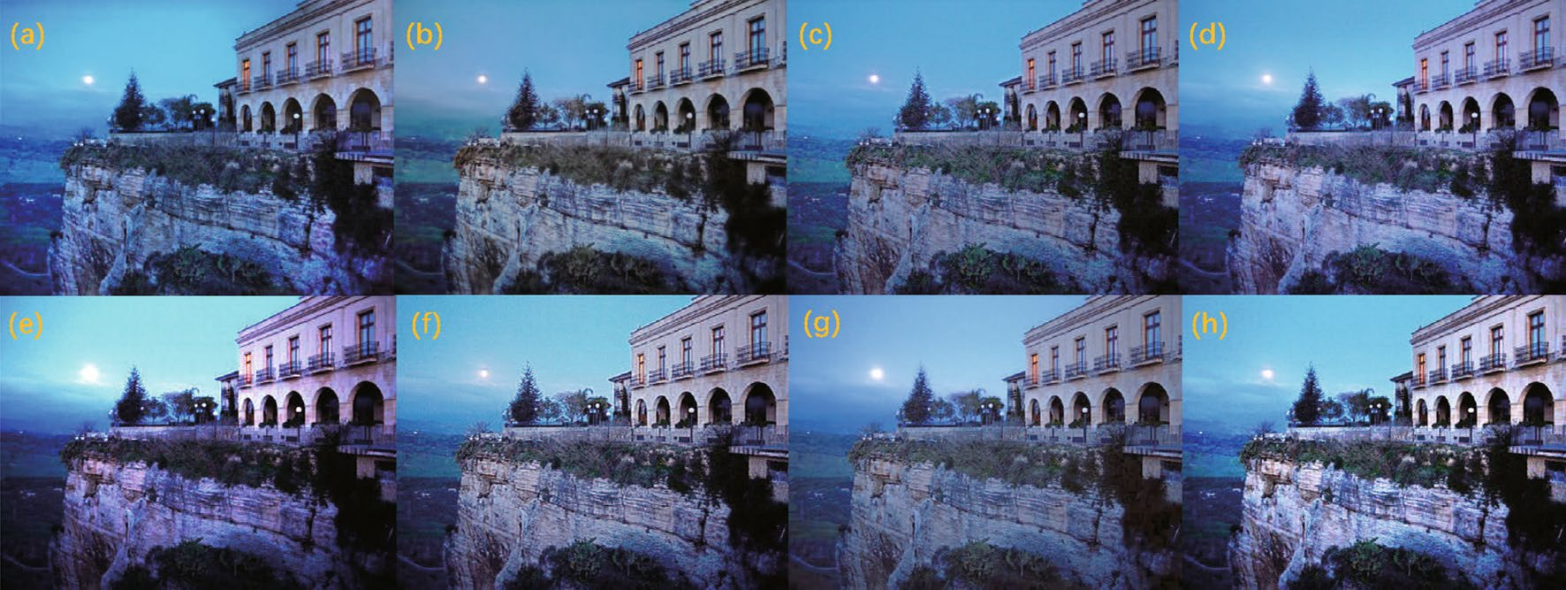
Test result display on the DICM dataset: (**a**) EnlightenGAN; (**b**) kinD; (**c**) RUAS; (**d**) ZeroDCE; (**e**) ZeroDCE++; (**f**) SCI; (**g**) Uretinex; (**h**) CUI-Net.

We conducted training and testing on the unpaired low-light enhancement datasets MEF^58^, VV, DICM^59^, and LIME^35^, with the qualitative results illustrated in Figs. 11, 12, 13, and 14 , respectively. As can be observed, our method effectively prevents overexposure across all four datasets, achieves a satisfactory enhancement of details, and restores realistic shadows and lightings. This can be observed, for instance, in the details of the tabletop, facial features, the flower cluster and door numbers, and the cliff and buildings.

The quantitative results are shown in Tables 3, 4, 5, and 6 .

**Table 3:** Quantitative test results on the MEF dataset.

Method	NIQE$\downarrow$	NIMA$\uparrow$	MUSIQ$\uparrow$	ILNIQE$\downarrow$
EnlightGAN	3.3953	4.585	62.0429	**23.4188**
KinD	3.8628	**4.8353**	63.0402	27.198
ZeroDCE	**3.3571**	4.8241	63.897	23.7894
ZeroDCE++	3.4655	4.7976	63.1217	23.5873
RUAS	3.8984	4.7827	61.1733	27.6975
SCI	3.7553	4.7878	63.0298	23.8953
Uretinex	3.8455	4.7728	*63.9931*	26.0306
CUI	*3.3277*	*4.8416*	**63.9748**	*23.0845*

The best results are highlighted in italic, and the second-best in bold.

**Table 4:** Quantitative test results on the VV dataset.

Method	NIQE$\downarrow$	NIMA$\uparrow$	MUSIQ$\uparrow$	ILNIQE$\downarrow$
EnlightGAN	**3.3504**	4.0177	59.3464	**23.6569**
KinD	3.5522	*4.7485*	61.1524	23.5128
ZeroDCE	3.8289	4.0979	**61.1756**	25.4865
ZeroDCE++	3.8855	4.0101	59.5025	26.0687
RUAS	6.0296	4.2009	53.8956	34.5125
SCI	3.8787	4.1887	60.9826	25.8835
Uretinex	4.0512	4.2036	60.9723	26.3003
CUI-Net	*3.1106*	**4.2671**	*61.6642*	*23.6009*

The best results are highlighted in italic, and the second-best in bold.

**Table 5:** Quantitative test results on the DICM dataset.

Method	NIQE$\downarrow$	NIMA$\uparrow$	MUSIQ$\uparrow$	ILNIQE$\downarrow$
EnlightGAN	**3.7664**	3.5572	54.7193	27.5723
KinD	3.9864	*3.8686*	56.304	27.1737
ZeroDCE	4.1108	3.6227	56.0174	**27.1695**
ZeroDCE++	4.0027	3.5537	55.7077	27.5014
RUAS	5.3462	3.7244	52.8156	34.6572
SCI	4.0816	3.7268	56.3253	27.7637
Uretinex	4.1505	3.7413	*57.9252*	27.4764
CUI-Net	*3.7589*	**3.7887**	**56.6143**	*26.968*

The best results are highlighted in italic, and the second-best in bold.

**Table 6:** Quantitative test results on the LIME dataset.

Method	NIQE$\downarrow$	NIMA$\uparrow$	MUSIQ$\uparrow$	ILNIQE$\downarrow$
EnlightGAN	**3.6648**	4.1859	58.5132	*26.5184*
KinD	4.4392	*4.6715*	58.4487	28.3259
ZeroDCE	3.6652	4.1888	59.3105	28.5489
ZeroDCE++	3.8375	4.3047	59.307	27.9016
RUAS	4.0366	4.1504	58.3871	28.7018
SCI	3.9357	4.135	59.5275	28.1087
Uretinex	4.3787	**4.4468**	*63.6361*	27.9216
CUI-Net	*3.6582*	4.2884	**59.716**	**27.8763**

The best results are highlighted in italic, and the second-best in bold.

From the tables, it can be observed that our method outperforms others in terms of quantitative results on unpaired low-light datasets, further demonstrating the robustness of our approach.

### Dark face detection

The DSFD^60^ face detection framework was utilized for the experiment, which adopts the SSD^61^ network structure and was trained on the WIDER FACE^62^ dataset. In the face detection experiment, results from different low-light enhancement methods were used as inputs to DSFD. Finally, we compared the AP (average precision) at different IoU thresholds. The test results are shown in Table 7, where CUI-Net achieved the highest AP values at IoU thresholds of 0.5 and 0.6 and the second-highest AP value at an IoU threshold of 0.7.

**Table 7:** AP (average precision) at IoU of 0.5, 0.6, 0.7 thresholds.

Method	IOU-0.5	IOU-0.6	IOU-0.7
Unenhanced	0.312537	0.294697	0.274237
EnlightGAN	0.397988	0.382441	0.363614
KinD	0.356347	0.342337	0.335421
ZeroDCE	**0.413927**	**0.397894**	*0.381572*
ZeroDCE++	0.343232	0.318213	0.299918
RUAS	0.297531	0.281332	0.274856
SCI	0.399491	0.384553	0.369657
Uretinex	0.406176	0.388785	0.366965
CUI-Net	*0.415792*	*0.399355*	**0.379995**

The best result is marked in italic, and the second-best result is marked in bold.

**Figure 15:**
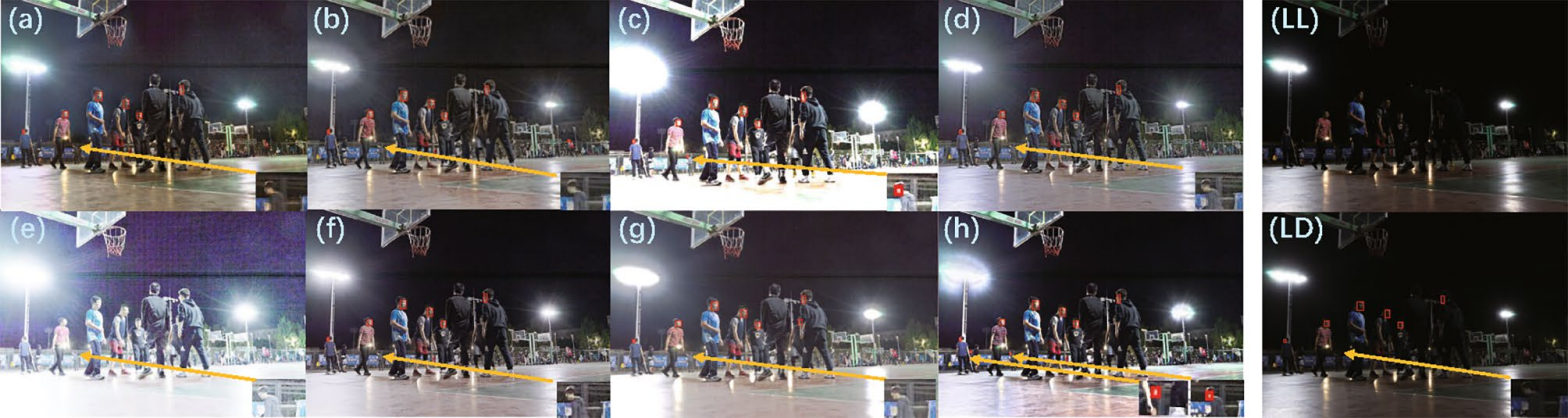
Results of dark face detection: (**a**) EnlightenGAN; (**b**) kinD; (**c**) RUAS; (**d**) ZeroDCE; (**e**) ZeroDCE++; (**f**) SCI; (**g**) Uretinex; (**h**) CUI-Net; (LL) Unenhanced low-light image as input; (LD) result of face detection directly on the Unenhanced low-light input image.

Figure 15 shows the detection results of different methods and adds the low-light input image (Fig. 15LL) and its face detection result (Fig. 15LD) for comparison. The lower right corner of each method’s result image is the corresponding magnified detail image. It can be seen that at an IoU threshold of 0.5, only RUAS (Fig. 15c) and CUI-Net (Fig. 15h) can detect the face in the area pointed by the arrow. EnlightenGAN (Fig. 15a), KinD (Fig. 15b), ZeroDCE (Fig. 15d), ZeroDCE++ (Fig. 15e), SCI (Fig. 15f), and Uretinex (Fig. 15g) failed to detect the face in the area pointed by the arrow. However, RUAS has serious overexposure, and the details on the ground cannot be seen clearly. CUI-Net not only can detect more face but also produces realistic enhancement effects, with better quantitative indicators than other SOTA methods.

### Low-light object detection

We trained the YOLOv3^63^ model on the ExDark object detection dataset and tested it on the ExDark validation dataset. YOLOv3 is a series of object detection frameworks and models pre-trained on the COCO dataset^64^. Unlike face detection experiments, we fine-tuned the YOLOv3 pre-trained model for object detection, i.e., we retrained the object detection model to evaluate the enhancement effects of all methods. Table 8 shows the quantitative results among different methods. CUI-Net achieved the best mAP values in both $mAP_{0.5:0.95}$ and $mAP_{0.5}$.

**Table 8:** Quantitative results of object detection on the ExDark dataset.

Method	$mAP_{0.5:0.95}$	$mAP_{0.5}$
BaseLine	**0.355**	0.64
EnlightenGAN	0.354	0.636
KinD	0.348	0.633
ZeroDCE	0.353	0.637
ZeroDCE++	0.324	0.605
RUAS	0.323	0.589
SCI	**0.355**	**0.642**
Uretinex	0.35	0.634
CUI-Net	*0.357*	*0.65*

The best results are marked in italic, and the second-best results are marked in bold.

**Figure 16:**
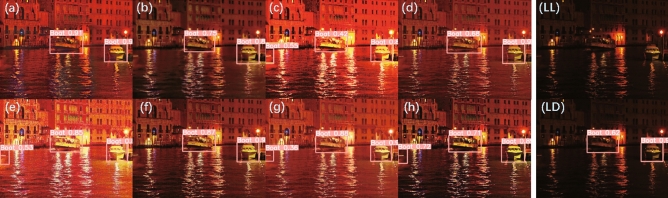
Experimental results of object detection on the ExDark dataset: (**a**) EnlightenGAN; (**b**) KinD; (**c**) RUAS; (**d**) ZeroDCE; (**e**) ZeroDCE++; (**f**) SCI; (**g**) Uretinex; (**h**) CUI-Net; (LL) Unenhanced low-light image as input; (LD) result of object detection directly on the Unenhanced low-light input image.

The experimental results were obtained by performing object detection on low-light images after being enhanced by various SOTA algorithms. The baseline is object detection directly on the unenhanced low-light images. The specific detection results object detection on the low-light image (Fig. 16LL) are shown in Fig. 16, Only RUAS (Fig. 16c), ZeroDCE++ (Fig. 16e), Uretinex (Fig. 16g), and CUI-Net (Fig. 16h) can recognize the most targets. EnlightenGAN (Fig. 16a), KinD (Fig. 16b), ZeroDCE (Fig. 16d), SCI (Fig. 16f), and baseline(Fig. 16LD) did not detect the targets completely. The overall average confidence values of RUAS, ZeroDCE++ and Uretinex are lower than CUI-Net. In addition, the main reason why RUAS and ZeroDCE++ have lower mAP values in Table 8 is due to the overexposure problem. However, CUI-Net found a good balance and was able to avoid the overall lower mAP scores caused by overexposure.

### Low-light semantic segmentation

We evaluated the performance of all segmentation methods on the ACDC low-light semantic segmentation dataset using the DeepLab-V3+^65^ model with pre-training and fine-tuning mode. The pre-trained model was trained on the Cityscape dataset^66^. Table 9 shows the mIoU values for multiple categories and the overall average among different low-light enhancement methods. CUI-Net achieved the best mIoU score among the six segmentation targets and was the second-best method among the seven segmentation targets. It outperformed the second-best method by 4.5 in the wall category, 1.9 in the traffic light category, and 6.6 in the motorcycle category. The overall average mIoU value was 2.8 higher than the second-best method.

**Table 9:** Shows the mIoU values for multiple categories average among different low-light enhancement methods.

Method	RO	SI	BU	WA	FE	PO	TL	TS	VE	TE	SK	PE	RI	CA	TR	MO	BI	mIoU
Base	89.3	58.5	62.1	26.6	25.5	2.5	42.4	36.3	33.8	12.6	42.7	33.6	*9.9*	62.4	66.6	**5.8**	44.7	34.5
EnGAN	92.1	*96.1*	*77.2*	35.8	33.3	49.2	53.5	43.5	**68.7**	9.7	81.6	25.4	6.9	63.2	83.8	2.7	**45.6**	**44.3**
KinD	90.1	63.5	73.9	33.5	26.7	*53.6*	**58.9**	**43.8**	67.3	6.8	*81.8*	28.4	**7.5**	52.3	69.9	3.3	30	41.6
ZeroDCE	91.6	68.7	77.2	35.9	*40.1*	45	31.3	40.6	68.8	9.7	80.6	*34.7*	5.3	*69.8*	*87.7*	2.7	36.9	43.5
DCE++	88.9	58.1	73.5	25.9	36.1	42.5	28.7	32	62.6	*16.7*	75.8	21.7	1.4	53.9	73.1	3.4	34.4	38.4
RUAS	88.3	61.7	75	34.5	32.1	42.8	45.5	32.2	65.4	8.7	80	15.8	0.5	56.8	75.8	1.6	25.5	39.1
SCI	89.6	65.5	76.5	**39.3**	38.7	41.8	54.5	41.9	67.1	7.8	79.2	32.7	7.2	44.9	84.5	2.7	17.4	41.6
Ureti	**92.4**	71.5	76.3	35.2	32.6	**50.3**	53.4	43.9	67	8	81.6	30.2	6.6	**67.2**	70.6	2.3	31.3	43.2
CUI	*94.1*	**74.2**	**77.2**	*43.8*	**39.4**	47.5	*60.8*	*48.3*	*68.9*	**14.1**	**81.6**	**33.7**	6.6	64.3	**84.5**	*12.4*	44.4	*47.1*

The symbol set {RO, SI, BU, WA, FE, PO, TL, TS, VE, TE, SK, PE, RI, CA, TR, MO, BI} represents {road, sidewalk, building, wall, fence, pole, traffic light, traffic sign, vegetation, terrain, sky, person, rider, car, train, motorcycle, bicycle}. CUI-Net had the highest mIoU value for four segmentation targets.

Table 10 shows the mAcc values for multiple categories average among different low-light enhancement methods. CUI-Net achieved the highest mAcc values for five segmentation targets, with 12.7 higher than the second-best method in the motor category and 22.9 higher in the rider category. CUI-Net also obtained the second-highest mAcc value for four segmentation targets, with an overall mAcc value 5 higher than the second-best method.

**Table 10:** Shows the mAcc values for multiple categories average among different low-light enhancement methods.

Method	RO	SI	BU	WA	FE	PO	TL	TS	VE	TE	SK	PE	RI	CA	TR	MO	BI	mAcc
Base	92.1	69.8	71.9	36.8	29.3	85.3	73.3	**60.0**	36.1	22.6	45.7	**43.9**	14.4	74.9	87.2	**6.0**	54.0	50.2
EnGAN	96.2	82.4	86.8	47.2	41.9	69.6	**76.4**	51.7	**84.1**	11.1	91.8	29.4	14.5	*84.5*	93.4	2.9	*66.3*	57.2
KinD	95.7	70.5	86.8	45.7	31.9	73.4	71.7	54.4	79.1	8.7	92.9	31.4	15.2	**83.7**	79.5	3.8	32.3	53.2
ZeroDCE	94.6	*85.5*	87.6	43.7	46.1	*76.8*	**80.2**	53.0	*86.1*	12.2	88.0	40.6	11.6	80.5	**96.0**	3.1	48.1	**57.4**
DCE++	95.5	69.8	85.6	32.7	*55.5*	72.3	72.9	46.8	77.1	*34.9*	85.0	24.2	3.5	70.1	87.2	5.2	49.7	53.8
RUAS	91.7	85.3	*90.9*	48.0	38.9	73.5	57.8	38.4	73.0	11.4	**93.5**	19.0	0.9	71.7	79.4	1.8	28.3	50.2
SCI	**96.4**	78.0	**88.2**	*59.4*	44.2	**75.4**	72.1	58.2	80.8	10.1	86.4	37.9	**15.4**	81.8	*96.5*	2.9	18.1	55.7
Ureti	95.1	**85.4**	87.4	42.6	37.3	61.8	68.5	56.4	81.3	9.7	*94.7*	34.5	9.8	78.1	94.6	2.7	34.3	54.1
CUI	*96.9*	84.3	87.8	**56.5**	**48.5**	74.3	76.1	*65.4*	82.2	**23.8**	90.8	*48.2*	*38.3*	79.2	95.5	*18.7*	**55.9**	*62.4*

The symbol set {RO, SI, BU, WA, FE, PO, TL, TS, VE, TE, SK, PE, RI, CA, TR, MO, BI} represents {road, sidewalk, building, wall, fence, pole, traffic light, traffic sign, vegetation, terrain, sky, person, rider, car, train, motorcycle, bicycle}. CUI-Net had the highest mAcc value for four segmentation targets.

**Figure 17:**
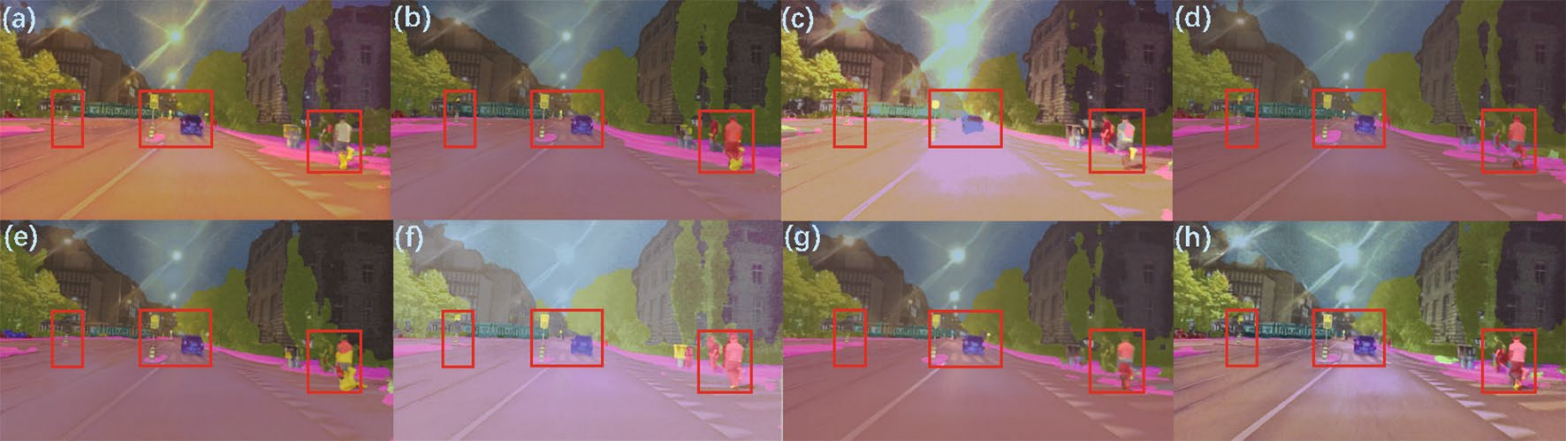
Segmentation results on the ACDC dataset: (**a**) EnlightenGAN; (**b**) kinD; (**c**) RUAS; (**d**) ZeroDCE; (**e**) ZeroDCE++; (**f**) SCI; (**g**) Uretinex; (**h**) CUI-Net.

Figure 17 shows the overlaid results of semantic segmentation masks and enhanced images on the ACDC dataset. Overall, RUAS (Fig. 17c) and SCI (Fig. 17f) exhibited overexposure. EnlightenGAN (Fig. 17a), KinD (Fig. 17b), ZeroDCE (Fig. 17d), ZeroDCE++ (Fig. 17e), Uretinex (Fig. 17g), and CUI-Net(Fig. 17h) methods showed no significant differences, but for nighttime semantic segmentation applications, attention to detail is particularly important, such as timely segmentation of pedestrians traffic signs on the road to avoid serious accidents during nighttime autonomous driving.

**Figure 18:**
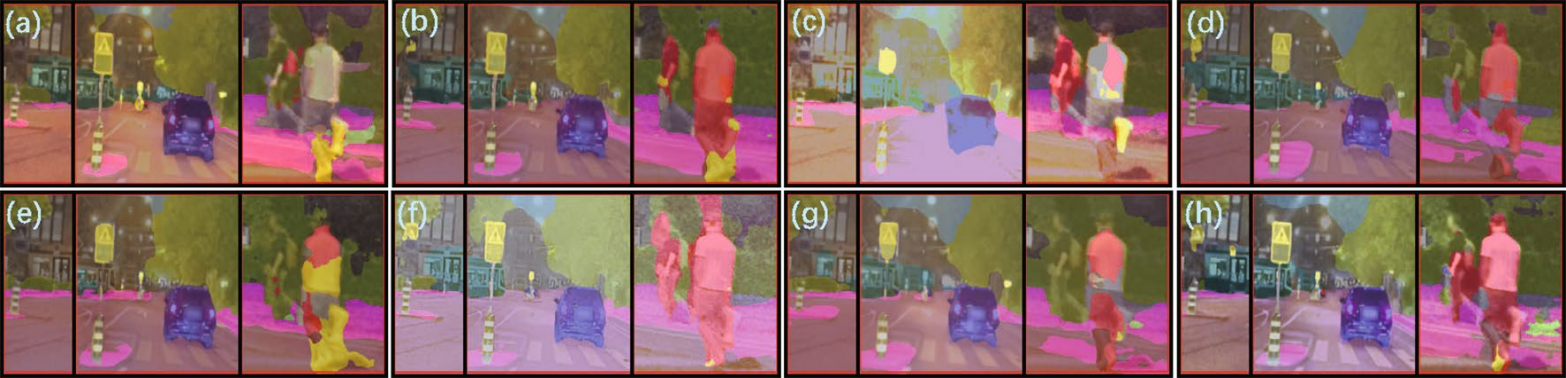
Enlarged details of the red boxes in Fig. 17: (**a**) EnlightenGAN; (**b**) KinD; (**c**) RUAS; (**d**) ZeroDCE; (**e**) ZeroDCE++; (**f**) SCI; (**g**) Uretinex; (**h**) CUI-Net;

**Figure 19:**
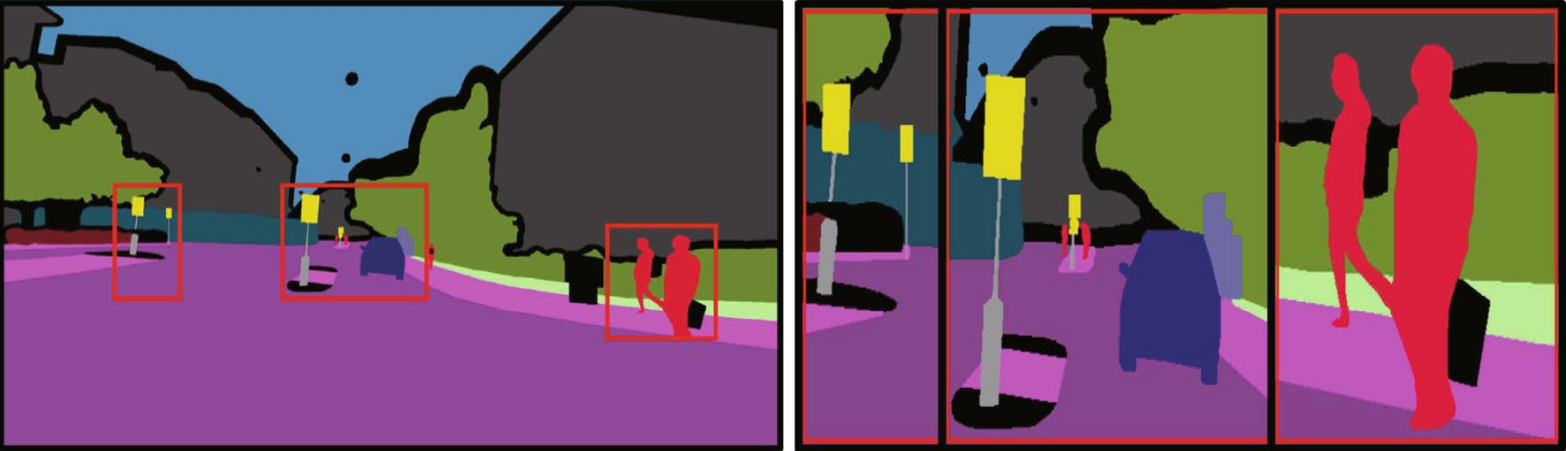
Left: Ground truth; Right: Image of zoomed-in details corresponding to the red area in the ground truth image.

The local detailed semantic segmentation results for each method corresponding to the red box in Fig.  17 are shown in Fig.  18. Comparing with the ground truth in Fig. 19, for the first red box region, which contains two traffic signs, EnlightenGAN (Fig. 18a), KinD (Fig. 18b), RUAS (Fig. 18c), ZeroDCE++ (Fig. 18e), and Uretinex (Fig. 18g) failed to segment both traffic signs, while ZeroDCE (Fig. 18d) and SCI (Fig. 18f) only recognized the left traffic sign. However, CUI-Net (Fig. 18h) was able to recognize both traffic signs. For the middle red box region, which contains two pedestrians and two traffic signs, only ZeroDCE++ (Fig. 18e) and Uretinex (Fig. 18g) recognized both traffic signs, while our CUI-Net (Fig. 18h) recognized an additional pedestrian. For the right red box region, which contains two pedestrians, only KinD (Fig. 18b), SCI (Fig. 18f), and CUI-Net (Fig. 18h) were able to segment both pedestrians well. In addition, for the pedestrian crossing category that does not exist in the ACDC dataset, it can be seen from Fig. 17 that CUI-Net has the most obvious enhancement effect, which may play a role in nighttime safety autonomous driving tasks. Clearly, CUI-Net has some potential in nighttime semantic segmentation tasks.

### Ablation study

To verify whether the network structure of the enhancement module in CUI-Net can improve the model’s enhancement ability, we conducted four ablation experiments on the LSRW dataset for training and testing, and evaluated the quality of the enhanced images using SSIM, PSNR, and LPIPS.

Firstly, to verify whether Adan and StarReLu can accelerate the convergence of the model, we choose to train for 50 epochs. The results obtained are shown in Table 11, where it can be observed that replacing GeLu with StarReLu and Adam with Adan can lead to better results in a smaller number of epochs.

**Table 11:** Ablation experiment of replacing GeLu and Adam with StarReLu and Adan.

Component	SSIM$\uparrow$	PSNR$\uparrow$	LPIPS$\downarrow$
Gelu & Adam	0.4305	16.264	0.333
StarRelu & Adan	0.4521	16.3019	0.3223

Secondly, to verify whether the network structure design of the enhancement module is effective, we replaced the five modules in the overall network with a full CNN module, a full Transformer module, and the three Transformer modules and two CNN modules of CUI-Net for experimental analysis. The results obtained are shown in Table 12, and the network structure of CUI-Net can achieve better performance.

**Table 12:** Replacing the five modules used in the original CUI-Net with different ones.

Component	SSIM$\uparrow$	PSNR$\uparrow$	LPIPS$\downarrow$
CNN only	0.4378	15.6028	0.3279
Transformer only	0.445	15.9146	0.3166
The modules of CUI-Net	0.4521	16.3019	0.3223

Thirdly, to verify whether MDSA and CGFN can improve the model’s enhancement ability, we selected MDTA and GDFN in Restormer for ablation study. The results are shown in Table 13, and both MDSA and CGFN can improve the performance of the model.

**Table 13:** Ablation experiments were conducted to compare the network module used in the Transformer block of CUI-Net with MDTA and GDFN.

Component	SSIM$\uparrow$	PSNR$\uparrow$	LPIPS$\downarrow$
MDTA+GDFN	0.44	16.2994	0.3285
MDSA+GDFN	0.4478	16.4507	0.3361
MDTA+CGFN	0.4223	16.753	0.3253
MDSA+CGFN	0.4521	16.3019	0.3223

Finally, an ablation study was conducted on the sparse attention operation on channels in the MDSA module of CUI-Net. The results are shown in Table 14. The $Topk\_normal$ operation is the usual sparse attention operation where all attention weights except for the *TopK* are set to zero. In contrast, the $Top\_CUI$ operation used in CUI-Net reduces the attention weights of the channels obtained by *TopK* to a very low value. The results of the ablation study indicate that the sparse attention on channels used in CUI-Net contributes to achieving better enhancement results.

**Table 14:** Perform an ablation experiment comparing the usual sparse attention mechanism with the sparse attention mechanism used in the CUI-Net network.

Component	SSIM$\uparrow$	PSNR$\uparrow$	LPIPS$\downarrow$
Topk_normal	0.4509	15.5529	0.3235
Topk_CUI	0.4521	16.3019	0.3223

## Conclusion

In this paper, we propose a CUI-Net framework consisting of an enhancement module and an auxiliary module, which can achieve differential enhancement of low-light and highlight regions in low-light environments. In the enhancement module, an efficient low-light enhancement Transformer and CNN network are introduced to enhance low-light images by acquiring global pixel information. In the auxiliary module, a lightweight CNN network is designed to assist the enhancement module to converge better and correct lighting effects. Quantitative analysis and qualitative comparison of CUI-Net with other state-of-the-art low-light image enhancement methods were conducted on two public low-light datasets, demonstrating the effectiveness of the proposed method. Furthermore, the practicality of the method was further verified through high-level vision tasks, namely low-light object detection, dark face detection, and nighttime semantic segmentation.

## Data Availability

The MIT dataset used during the current study are available in the https://data.csail.mit.edu/graphics/fivek/. The LSRW dataset used during the current study are available in the https://github.com/JianghaiSCU/R2RNet. The DarkFace dataset used during the current study are available in the https://flyywh.github.io/CVPRW2019LowLight/. The ExDark dataset used during the current study are available in the https://github.com/cs-chan/Exclusively-Dark-Image-Dataset. The ACDC dataset used during the current study are available in the https://acdc.vision.ee.ethz.ch/. The MEF, VV, DICM and LIME datasets used during the current study are available in the https://github.com/Li-Chongyi/Lighting-the-Darkness-in-the-Deep-Learning-Era-Open/.
